# Accurizing the Cause of Stillbirth Reviewed Under Maternal and Perinatal Death Surveillance and Response System Through Minimally Invasive Tissue Sampling: A Case Report

**DOI:** 10.1002/ccr3.70979

**Published:** 2025-10-02

**Authors:** Nuwadatta Subedi, Junu Shrestha, Sabita Paudel, Sunita Ranabhat, Dela Singh

**Affiliations:** ^1^ Department of Forensic Medicine Gandaki Medical College Teaching Hospital and Research Center Pokhara Nepal; ^2^ Department of Obstetrics and Gynaecology Manipal College of Medical Sciences Pokhara Nepal; ^3^ Department of Pharmacology Gandaki Medical College Teaching Hospital and Research Center Pokhara Nepal; ^4^ Department of Pathology Gandaki Medical College Teaching Hospital and Research Center Pokhara Nepal; ^5^ Department of Obstetrics and Gynaecology Pokhara Academy of Health Sciences Pokhara Nepal

**Keywords:** maternal and perinatal death surveillance and response, minimally invasive tissue sampling, placental insufficiency, preeclampsia, stillbirth

## Abstract

Stillbirth is a public health challenge to low‐ and middle‐income countries, and the rate of stillbirths in Nepal is 15.7 per 1000 births. Stillbirths are reviewed under the maternal and perinatal death surveillance and response (MPDSR) system in Nepal, where the causes of deaths and preventive strategies should be identified, but there are constraints in identifying accurate causes of stillbirths. The existing MPDSR system lacks a depth review of perinatal deaths, just relying on the clinical and demographic information; thus, the accurate causes are left unidentified on several occasions. We incorporated minimally invasive tissue sampling (MITS) in a case of stillbirth reviewed under MPDSR as a part of a pilot project for the first time in Nepal in the MPDSR system. Histopathology of lung tissue samples identified meconium aspiration, and the placental examination identified maternal vascular malperfusion, which was a sequel of preeclampsia. Based on the placental and lung tissue findings, the main fetal cause was identified as intrauterine hypoxia. Along with the clinical information, MITS and placental examination are useful to establish accurate causes of stillbirth, reviewed under the MPDSR system, and to develop targeted strategies for the prevention of future deaths.

**Summary**

- Minimally invasive tissue sampling and placental examination, when added to the existing MPDSR system, are useful to explore the causes of stillbirth accurately.
- The pathological events underlying the causes of stillbirth can be precisely explained, which can be utilized to plan for preventing future perinatal deaths.

## 1 Introduction

Stillbirth is one of the public health challenges in developing countries as indicated by the prevalence of approximately 98% of stillbirths globally occurring in low‐ and middle‐income countries (LMICs) [1]. The rate of stillbirth in Nepal in 2021 was approximately 15.7 per 1000 total births; [2] however, the rates are highly variable in different settings [3]. Every Newborn Action Plan (ENAP) aims for all nations to lower the stillbirth rate to 12 or fewer per 1000 total births by the year 2030 [4]. This goal is in line with the Sustainable Development Goals (SDGs), particularly SDG 3.2, which focuses on eliminating preventable deaths among newborns and children under the age of five by 2030 [5]. One of the important ways to minimize the rate of stillbirth is to explore the accurate cause and to develop action plans to prevent such conditions. Maternal and Perinatal Death Surveillance and Response (MPDSR) is implemented in Nepal since 2015 on the background of the existing maternal death review (MDR) system [6]. This is a program to explore the causes of maternal and perinatal deaths and to develop responses learning from each death in order to develop preventive mechanisms [7].

However, there are several challenges in the effective implementation of the program and one of them is the difficulty in determining the accurate causes of death. In the latest report by the Family Welfare Division of Nepal, the cause of death was not determined in 57% of antepartum stillbirths and 43% of intrapartum stillbirths [8]. The problem in determining the cause of death might be due to the lack of fetal autopsy and the unavailability of a protocol for placental examination. Fetal autopsy is not a regular process in Nepal, and the policy is not mandatory for placental investigation, though some institutes have started on their own. The other challenge seems to be the inadequate review of the deaths, even with the available information. The reason could be the lack of training for the data enumerators and reviewers. In order to overcome such challenges, we are implementing a project to utilize minimally invasive tissue sampling (MITS) in perinatal deaths reviewed under the MPDSR in Kaski District of Nepal [9]. MITS is a procedure to retrieve biological samples from the deceased and perform microbiological and histopathological tests to determine the accurate causes of death. The implementation of MITS into the stillbirths and children of age under 5 years in Child Health and Mortality Prevention Surveillance (CHAMPS) network was successful in identifying at least one cause of death in 98% of 933 cases. The use of MITS also enabled the characterization of contributory conditions and broadened the spectrum of events leading to fetal and child death [10]. “Perinatal MITS Nepal” is a project implemented in hospitals undergoing the MPDSR program in Kaski district of Nepal. The perinatal deaths reviewed in MPDSR are also enrolled in MITS, where sample retrieval is performed after obtaining consent. Then, detailed clinical information is gathered, laboratory examinations are performed, and finally, a panel of experts determines the cause of death.

## 2 Case History/Examination

A 32‐year‐old gravida 3 para 1 abortion 1 presented to one of the tertiary care centers implementing the MPDSR program in Kaski district of Nepal at her gestational age (GA) of 32 weeks. The GA was calculated based on a certain last menstrual period. She had undergone four antenatal visits at a district hospital. She had taken two doses of tetanus and diphtheria (TD) vaccine as per the protocol [11]. She had initially presented to a district hospital 40 km away from the tertiary referral center along a national highway with complaints of headache and absent fetal movements on 15th January 2024. She was diagnosed with hypertension, with a blood pressure of 160/100 mmHg, and was started on Methyldopa 250 mg and referred to the tertiary center for further management. She was not diagnosed with hypertension in her previous antenatal visits. She had never undergone screening for gestational diabetes in the antenatal checkups. When she reached the referral center, her blood pressure was high, the uterus was only 28 weeks size, and fetal heart sound was not heard. Diagnosis of intrauterine fetal death was confirmed with ultrasound. The hemoglobin level of the mother was 12.8 g/dL, and the urine examination for albumin showed a positive result. Random blood sugar was 138 mg/dL, HbA1c 6%, LDH 258.55 U/L, and serological tests for HIV, HBS Ag, and VDRL were negative.

She was diagnosed as G3 P1 + 1 at 32 weeks of gestation with intrauterine fetal death with preeclampsia with impaired glucose tolerance and a history of previous cesarean section. Mechanical induction of labor was performed followed by administration of oxytocin. The stillborn fetus was delivered vaginally after 6 h of induction. The intrapartum and postpartum periods were uneventful. The mother recovered following delivery and was discharged on the second postpartum day. The family members were approached for consent for the clinical information, MITS procedure, and placenta examination as per the protocol of the project “Perinatal MITS Nepal” [9], and then consented to the information and both procedures. MITS was performed 12 h after expulsion of the fetus. The fetus was Grade II macerated, weighed 1 kg, and had a head‐to‐toe length of 33 cm. The head circumference was 28 cm, which was near the 10th centile (28.21 cm) for the GA of 32 weeks based on the intergrowth criteria [12]. The intergrowth percentile using weight and GA was 0.0, and using weight, GA plus the fetal length was 3.18, and both were below the cutoff value of the 10th percentile; thus, a diagnosis of small for gestational age was made [13].

**FIGURE 1:**
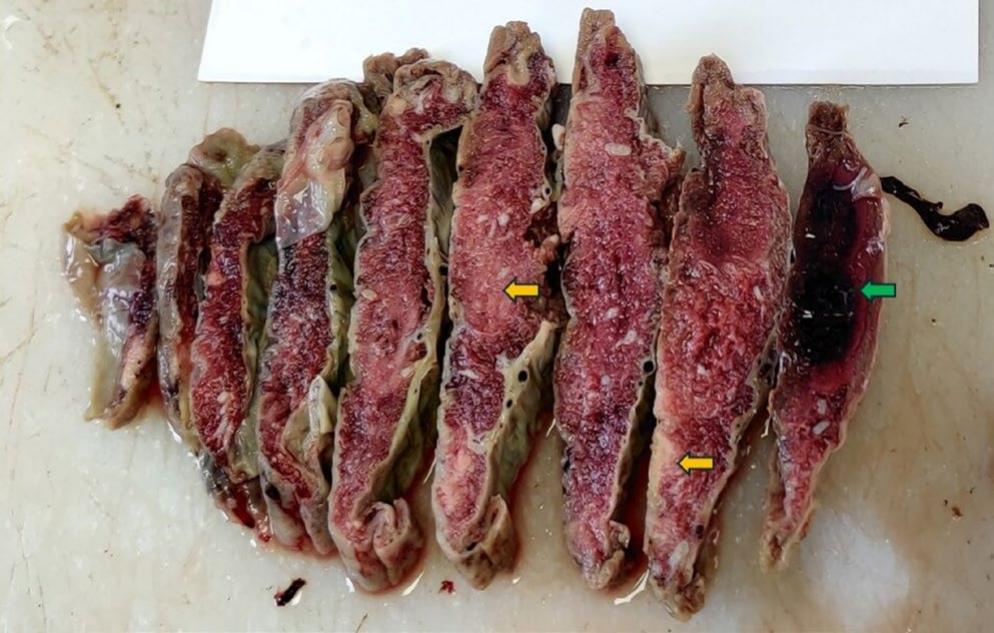
Gross picture of placenta showing whitish area suggestive of infarction (yellow arrows) and area of hematoma (green arrow).

Placenta examination was performed as per the Amsterdam protocol [14]. There were no significant pathological findings on gross examination; the maximum length was 9 cm and the breadth 8 cm. The weight of the trimmed placenta, removing the membranes and the umbilical cord, was 150 g, and the length of the umbilical cord was 40 cm. The tissue samples were collected by the standard MITS procedure [15] Samples from the right and the left lungs were obtained for microbiological tests. The brain, right lung, left lung, and liver tissue samples were obtained for histopathological examinations. The blood sample was retrieved by direct puncture to the heart. The detailed procedure of sampling, laboratory examinations, and cause of death determination is described in the published study protocol of “Perinatal MITS Nepal” [9] The blood culture, culture of the right and left lung tissues, and culture of the placenta and the membrane separately showed no growth of microorganisms. The screening for rubella, toxoplasmosis, cytomegalovirus, and herpes simplex (TORCH) from the fetal blood was negative. Likewise, malaria antigen and *Treponema pallidum* hemagglutination (TPHA) tests were negative. The serological tests for HIV and HBsAg were also negative. The gross examination of the placenta after fixation and sectioning demonstrated whitish areas suggestive of infarction, both fresh and old, and areas of hematoma (Figure 1).

**FIGURE 2:**
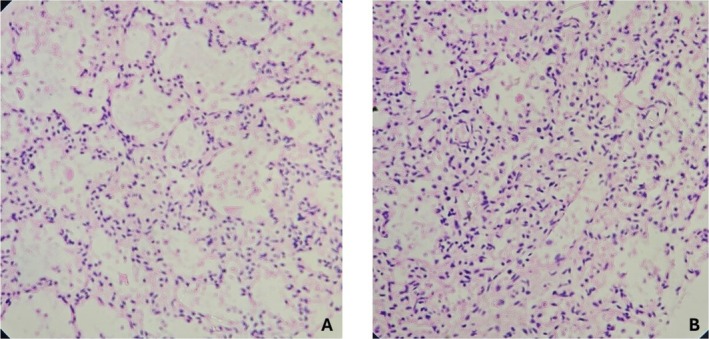
H&E 40×: Normal pneumocytes lining the wall and alveolar space filled with proteinaceous material and few anucleated squames in the right lung (A) and the left lung (B) suggestive of meconium aspiration.

**FIGURE 3:**
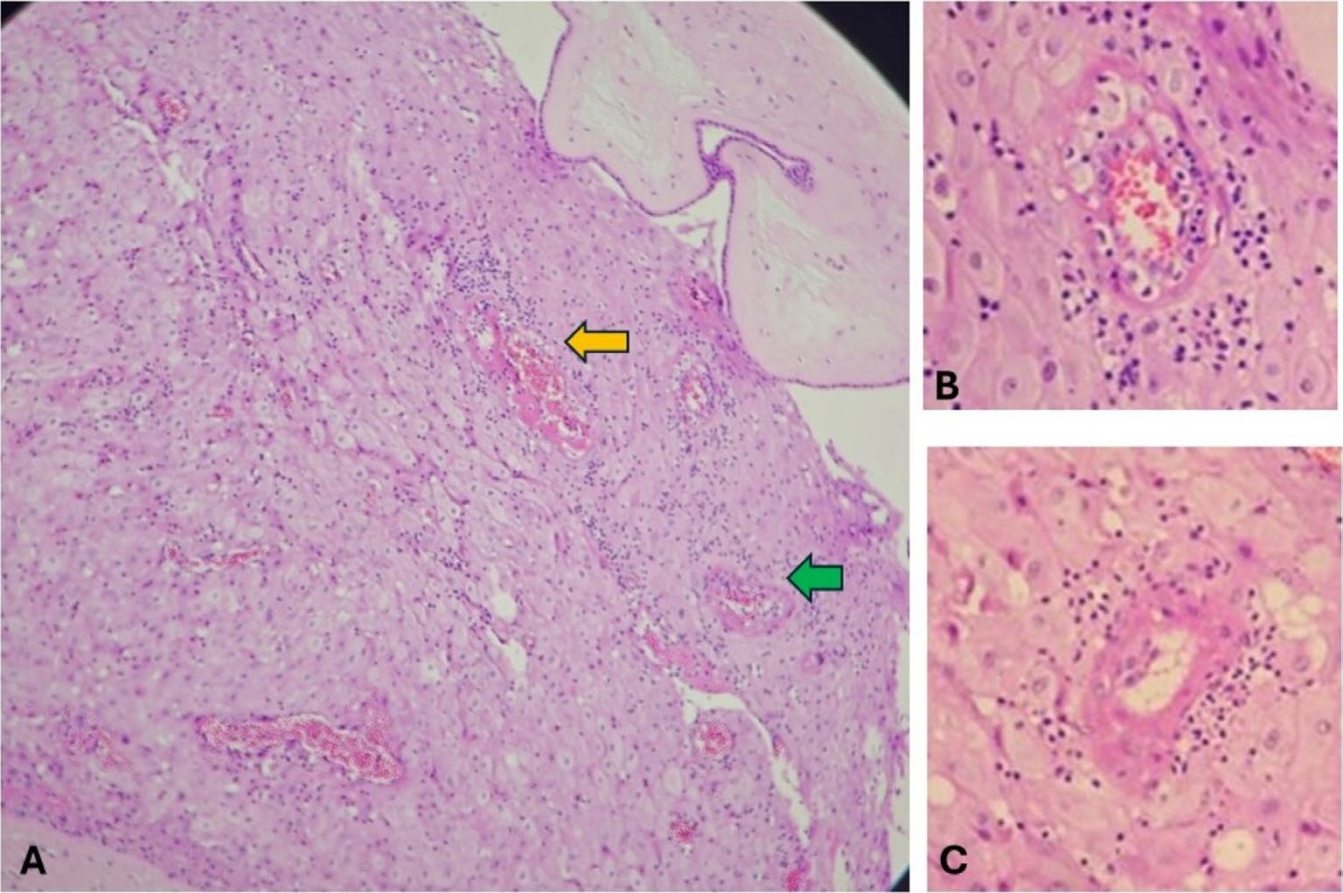
(A) H&E 4×: Section from the placental disc shows decidual vessels with features of fibrinoid necrosis of the wall and deposition of foamy macrophages in the wall (A). A magnified view (40×) of the lesions in the yellow and green arrows is depicted in images (B, C) respectively, suggesting decidual arteriopathy.

**FIGURE 4:**
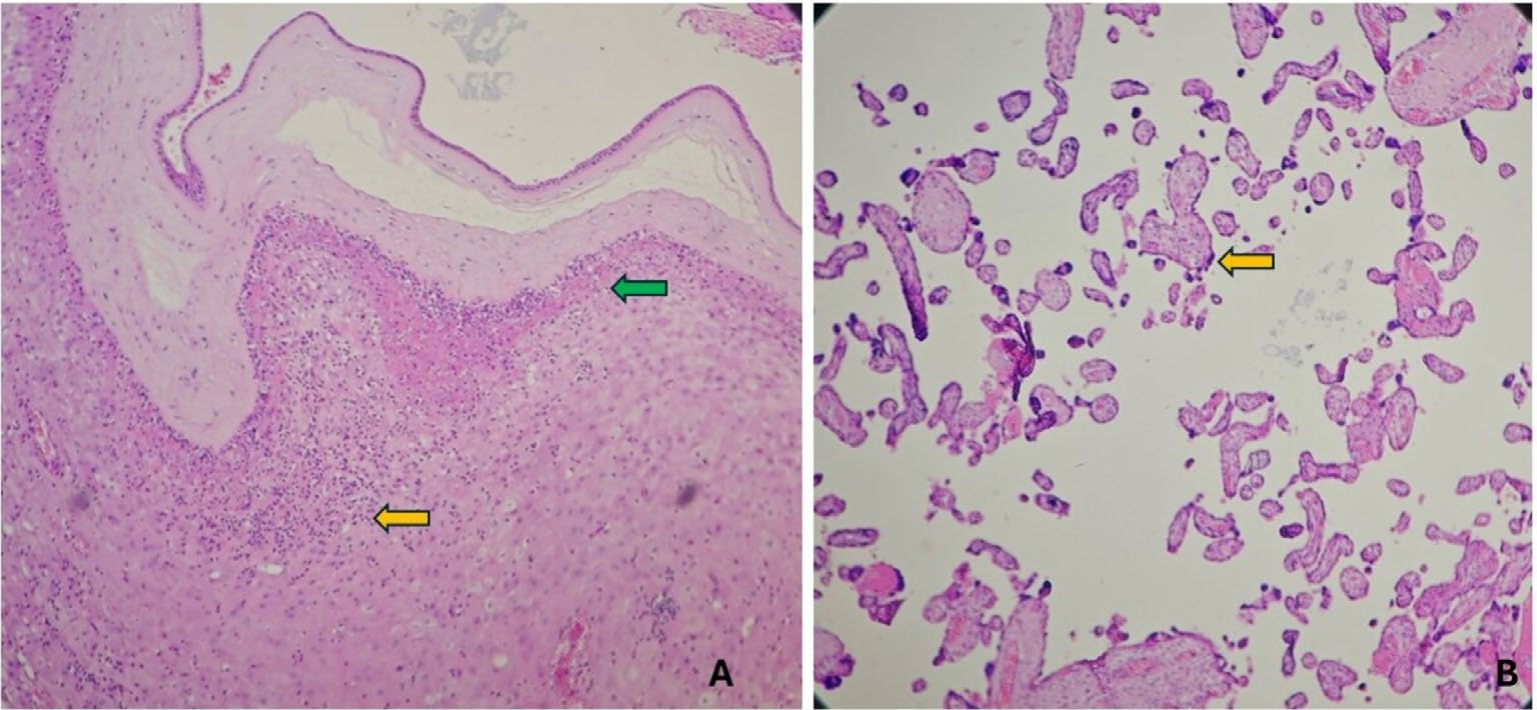
(A) H&E 4×: Section from membrane shows dense neutrophilic infiltration with some foci showing neutrophilic abscess revealed by yellow and green arrows, suggestive of deciduitis. Amnion and chorion appear unremarkable. (B) H&E 10×: Section from disc shows less crowded tertiary villi and appear small and angulated (yellow arrow), diagnostic of distal villous hypoplasia.

The histological examination of the right and the left lungs showed alveolar spaces filled with proteinaceous material and few anucleate squames, evidence of meconium aspiration due to intrauterine fetal distress (Figure 2A,B). Sections from the fetal and maternal cord appear unremarkable with two arteries and a single vein wrapped in Wharton's jelly‐like substance. Similarly, sections from the disc demonstrated deposition of foamy macrophages in the wall of the decidual vessels. There was acute atherosis and fibrinoid necrosis of the wall of the blood vessels, suggestive of decidual arteriopathy as depicted in Figure 3. Sections from the membrane roll demonstrated dense neutrophilic infiltration with some foci showing neutrophilic abscess suggestive of deciduitis; however, the amnion and chorion were unremarkable as revealed in Figure 4A. There was a decrease in the number of chorionic villi and foci with infarcted villi (Figure 4B). One focus of infarction hematoma was also seen. The diagnosis of maternal vascular malperfusion with distal villous hypoplasia and decidual arteriopathy was established based on the placental histopathological findings.

A summary of the case was prepared, compiling all the information, and it was subjected to review from the cause of death panel of the project. The cause of death was determined according to the protocol of the International Classification of Diseases‐Perinatal Mortality (ICD PM), which identifies both fetal causes and maternal causes. The fetal cause is categorized as main fetal and other fetal causes, and the maternal causes are also classified as the main maternal and other maternal causes [16].

## 3 Conclusion and Results

Taking the available maternal information, gross examination findings of the fetus, and the MITS‐informed laboratory findings into consideration, intrauterine hypoxia was established as the main cause of fetal death. Intrauterine hypoxia was diagnosed based on the evidence of placental insufficiency demonstrated by the histological evidence of placental insufficiency [17], as well as the clinical evidence of preeclampsia in the mother. The evidence of meconium aspiration by histological evidence also adds to the causation of fetal distress; however, it is not conclusive as it suggests the consequence of hypoxia rather than the cause [18] The other fetal cause of death was confirmed as small for gestational age. The main maternal cause of fetal death was preeclampsia, as suggested by the clinical evidence of hypertension and the presence of proteinuria, and the other maternal cause was identified as placental insufficiency. Placental insufficiency is the effect of preeclampsia, and it is suggested by the lesions of maternal vascular malperfusion, such as villous hypoplasia, foci with infarcted villi, and decidual arteriopathy, which enhanced the evidence to make a conclusive diagnosis [19].

## 4 Discussion

The accurate cause of death is necessary for effective response planning in the MPDSR system. Accurate cause will help to understand the causal chain of events which in turn will assist in planning and implementing measures to intervene. The specific cause of death was determined primarily on the basis of the histopathological findings of the placenta in this case. The evidence of meconium aspiration in the fetal lungs suggested fetal distress, most likely due to the intrauterine hypoxia caused by placental insufficiency. The placenta and lung samples are useful for the determination of the cause of death in stillbirths as depicted by Goldenberg et al. [20], while the other tissue samples, such as brain and liver, are least useful. The intrauterine growth restriction in this case is most likely due to the compromise of uteroplacental blood flow because of hypertension [21]. Hypertensive disorders are associated with an increased risk of maternal morbidity and mortality. Women who possess a history of hypertensive disorders during pregnancy, especially those who experience preeclampsia, are at an increased risk of developing cardiovascular disease in the future [22].

Therefore, early diagnosis and treatment of gestational hypertension are important to prevent the sequelae of events leading to fetal demise as well as to prevent maternal complications. Though the mother had undergone four antenatal checkups in a district hospital, hypertension was not identified earlier, suggesting the need for strengthening of quality of antenatal care in our setting. Utilizing MITS and placental examination, fetal asphyxia was identified as the most common cause of stillbirths, with maternal hypertension, particularly preeclampsia, as the leading maternal cause as presented by PURPOSe study from India and Pakistan [23]. Fetal autopsy plays an important role in exploring the diseases and conditions leading to stillbirth. A thorough fetal autopsy and placental examination, combined with detailed clinical data collection, are essential in assessing stillbirths and can significantly contribute to reducing the rate of unexplained stillbirths [24]. Clinical examination alone might not be sufficient to establish accurate causes as evidenced by the latest data from the Family Welfare Division of the Ministry of Health and Population, Nepal, demonstrating the inability to identify causes of more than half of stillbirths by MPDSR alone [8].

In the context where a complete fetal autopsy is not possible, MITS can be a valuable tool to accurately determine the causes of stillbirth. Together with the placental examination and laboratory examination of the tissue samples and specimens, it can have great value, as evidenced in this discussed case. In this case, the placental lesions, like the infarcts and hematoma, were identified by gross examination after fixation, whereas the histological lesions, including the decidual arteriopathy and distal villous hypoplasia, suggested fetal vascular malperfusion. The histological evidence of meconium aspiration in the lungs supported the intrauterine hypoxia, with the pathogenesis of placental insufficiency. Deciduitis, as diagnosed by the histological examination of the placental membranes, was not taken into consideration for the cause of death, as it was not backed up by other evidence of infection, such as the absence of growth of organisms in the culture of placental and lung tissues and the fetal blood.

The grade of deciduitis was also not high enough to back up the diagnosis. This could be explained as the sequel of intrauterine fetal death rather than the cause. Through the incorporation of MITS, the pathological events could be understood more accurately and it also aided in ascertaining the causes of death in the causal chain of events. In the Nepalese context and many other LMICs, the placental examination is not performed routinely, thus missing several pieces of information that could have been useful for the prevention of future stillbirths. Some hospitals have started placental investigations in Nepal at their own discretion, but policy is not mandatory in all cases. Also, the pathologists are also not well‐trained to perform placental examinations. Placental examination is not only important to unravel the causes of stillbirths but also to explore several conditions that can assist in improving future maternal and neonatal health; thus, it is recommended in cases with clinical indications [25]. Complete diagnostic autopsy (CDA) is the gold standard for determining the cause of death; however, it is not commonly performed in developing countries, including Nepal, unless there is a legal indication. In this context, MITS can be useful as it is less time‐consuming, causes less disfigurement, and is more acceptable to the family members, and can produce comparable results with the CDA [26].

The preventability of the stillbirth was assessed based on the guidelines laid out from the observation of a similar context [27], and this case was determined as potentially preventable. The improvement in the quality of antenatal care, coupled with the provision of quality clinical care, could have identified hypertension at an earlier stage and prevented the further pathological sequence to cause fetal demise. The provision of proper health education for the pregnant mother and her family, along with the health‐seeking behavior, could also have contributed to preventing the death.

As we are marching toward minimizing the rate of stillbirths and attaining the goals as laid out by the SDG, the identification of accurate causes is important in order to develop preventive strategies. Though the MPDSR system is an effective way of identifying the causes and circumstances of stillbirths and responding accordingly, it lacks its effectiveness mainly in determining the accurate cause of stillbirths because it only considers clinical and demographic information. Therefore, it is high time to include placental examinations and MITS to collect samples for further laboratory examinations to accurize the causes of stillbirths.

The incorporation of methods of MITS and placental examination increased the accuracy of the determination of causes of stillbirth. This case report highlights the importance of meticulous analysis of the case details of the mother, placental examination, and MITS of the stillbirth, and careful interpretation of the findings can help frame specific causes of death, which can further improve the MPDSR system and develop strategies to prevent future death.

The limitations of the case include the lack of molecular analysis of the samples, which could have further contributed to the identification of infectious agents, if any.

## Author Contributions

**Nuwadatta Subedi:** conceptualization, funding acquisition, methodology, project administration, supervision, validation, writing – original draft, writing – review and editing. **Junu Shrestha:** formal analysis, investigation, methodology, supervision, validation, writing – original draft, writing – review and editing. **Sabita Paudel:** conceptualization, formal analysis, investigation, methodology, project administration, writing – original draft, writing – review and editing. **Sunita Ranabhat:** data curation, formal analysis, investigation, methodology, validation, writing – original draft, writing – review and editing. **Dela Singh:** data curation, supervision, writing – original draft, writing – review and editing.

## Ethics Statement

Informed written consent was obtained from the legal relative of the deceased.

## Conflicts of Interest

The authors declare no conflicts of interest.

## Data Availability

Data sharing was not applicable to this article as no datasets were generated or analysed during this study.
